# Impact of different treatment plans on EQD_2_ for intracavitary brachytherapy of cervical cancer

**DOI:** 10.1186/s43046-021-00084-2

**Published:** 2021-10-04

**Authors:** Osman Vefa Gul, Gokcen Inan, Hamit Basaran

**Affiliations:** Department of Radiation Oncology, Faculty of Medicine, Selcuk University, Konya, Turkey

**Keywords:** EQD2, Cervical cancer, Brachytherapy

## Abstract

**Background:**

Cancer is one of the leading causes of death worldwide. According to GLOBOCAN estimates, there were 341,831 deaths from cervical cancer in 185 countries in 2020. The aim of this study was to compare equieffective dose (EQD_2_) at 2 Gy per fraction by using dose volume histograms (DVHs) derived from external beam radiotherapy (EBRT) and high-dose rate brachytherapy (HDR-BRT) treatment plans used in cervical cancer radiotherapy.

**Methods:**

Fifteen patients with stage IIB-IIIB cervical cancer were included in this retrospective study. Treatment with three-dimensional conformal radiotherapy (3D-CRT) or intensity-modulated radiotherapy (IMRT) was planned for all patients in 28 fractions, with a total of 50.4 Gy to be delivered to the whole pelvic region. After EBRT, manual optimization (MO) or inverse optimization (IO) HDR-BRT plans were created in 4 fractions with a total of 28 Gy. The plans obtained were grouped as IMRT + IO, IMRT + MO, 3DCRT + IO, and 3DCRT + MO by calculating EQD_2_s among these plans. D_90_, D_95_, and D_98_ values were compared in all plans for CTV_HR_ total EQD_2_. In addition, EQD_2_ values ​for critical organs at risk (OARs) such as rectum, bladder, small intestine, and sigmoid were compared in all plans for volumes of 2 cm^3^, 1 cm^3^, and 0.1 cm^3^, respectively.

**Results:**

There was no significant difference between the treatment groups in terms of CTV_HR_ D_90_ and CTV_IR_ D_90_ values; However, CTV_HR_ D_95_ (*p* = 0.000) and CTVHR D98 (*p* = 0.000) values ​were found to be better in IMRT + IO technique. The IMRT + IO technique provided better protection for 2 cm^3^, 1 cm^3^, and 0.1 cm^3^ volumes of OARs compared to other techniques.

**Conclusions:**

Considering all parameters such as CTV_HR_, CTV_IR_, rectum, bladder, small intestine, and sigmoid, combination of IMRT + IO treatment option was found to be significantly superior in total EQD2 calculations compared to other plans.

## Background

GLOBOCAN estimates of worldwide mortality and incidence for 36 cancers in 185 countries showed 614,127 new cases of cervical cancer and 341,831 deaths from this malignancy in 2020 [1]. In cervical cancer, in determining the treatment method, it is necessary to know the characteristics of the tumor as well as the mode and ways of its spread. The International Federation of Gynecology and Obstetrics (FIGO) staging system, which is mainly a clinic pathological staging system based on tumor size and its spread in the pelvis, is used. Radiotherapy is the standard approach in the treatment of locally advanced cervical cancer (LACC), and the success of radiotherapy depends on the use of brachytherapy (BRT) after external radiotherapy (EBRT), optimization of the dose received by the tumor and normal tissues, and the total duration of the treatment [2].

Today, three-dimensional conformal radiotherapy (3DCRT) and intensity-modulated radiotherapy (IMRT) are widely used in the external treatment of cervical cancer with technological possibilities [3, 4]. The 4-field technique is widely used in cervical cancer 3DCRT, but considering the radiation field size, this area covers important areas of organs at risk (OARs) such as the bladder, rectum, sigmoid, and small intestine. In the treatment plan created in the form of the pelvic box, it is aimed to deliver a total dose of 45–50 Gy to the lymphatics in 25–28 fractions [5–7]. IMRT provides potential benefit over 3DCRT for target area improvement and reduces normal tissue toxicity.

It is important to apply BRT after EBRT in cervical cancers. In today’s BRT, remote controlled afterloading applications are made. Since the treatment and care team are exposed to less radiation in afterloading BRT applications, remote controlled loading methods are widely used in many centers. Generally, Ir192 is preferred as a source in high-dose rate (HDR) applications [8]. Computed tomography (CT) and magnetic resonance imaging (MRI) compatible tandem-ovoid (TO) applicators have been developed. In the light of all these developments, European Society for Radiotherapy & Oncology (GEC-ESTRO) recommended high-risk clinical target volume (CTV_HR_) and intermediate- risk clinical target volume (CTV_IR_). For OARs, the dose taken by 2 cm^3^ of the volume (D_2cm_^3^), referenced the concepts of D_1cm_^3^ and D_0.1cm_^3^ [9].

To compare the different dose rates and fractionation schemes used in BRT and to make it possible to add BRT doses to EBRT, GEC-ESTRO, International Commission on Radiation Units and Measurements (ICRU) has encouraged the use of the concept of equivalent-effective doses. EBRT and BRT schemes are recalculated according to the linear quadratic (LQ) model and expressed as equieffective total dose (EQD_2_) as if it is applied with fractions of 2 Gy. By using the LQ model for the total EQD_2_, BRT’s EQD_2_ and EBRT’s EQD_2_ are calculated and summed up with each other. For radiobiological parameters, the tumor α/β is taken as 10 Gy and OAR α/β as 3 Gy. According to ICRU 89, the total EQD_2_ should be equivalent to 85 Gy for 90% (D_90_) of the CTV_HR_ volume and 65 Gy for CTV_IR_ D_90_. Also, the total EQD_2_ limits for D_2_cm^3^ of OARs should be as follows: 90 Gy for bladder and 75 Gy for rectum, sigmoid, and small bowel [10–13]. In HDR-BRT, the dose is controlled by changing the radiation dwell times at source dwell positions during the planning phase. In BRT planning, the optimization process through manual change of radiation dwell times or weights is called manual optimization (MO). In this method, the dose distribution that meets the criteria is tried to be obtained by trial and error method by constantly changing the dwell times. This process depends on the planner’s experience [14]. In BRT planning, the optimization process used to create the optimum plan in applications with a large number of resource dwell positions and targeted criteria is called inverse optimization (IO). By providing faster solutions in BRT planning, IO can provide the closest dose distributions to the desired one.

In this retrospective study, 3DCRT and IMRT plans were made for each one of the 15 patients with a diagnosis of cervical cancer with FIGO stage IIB and IIIB using a total of 50.4 Gy in 28 fractions for the whole pelvis. Then, MO and IO plans were made to CTV in 4 fractions so as to deliver 28 Gy in total. The created plans were grouped as IMRT + IO, IMRT + MO, 3DCRT + IO, and 3DCRT + MO by calculating EQD_2_s separately for each plan. The total EQD2 values obtained were dosimetrically evaluated in terms of CTV and OARs.

## Methods

### Patients

Cervical cancer patients with FIGO stages IIB to IIIB treated between February 2019 and March 2020 were included in this retrospective study. For this study, permission was obtained from the ethics committee of Selcuk University Faculty of Medicine, with the decision numbered 2020/424, dated September 30, 2020. Fifteen patients who underwent EBRT and intracavitary brachytherapy were included. Since the CTV and OAR values obtained for each technique were similar, the sample size was limited to 15 patients. In our clinic, the chemoradiotherapy protocol accepted simultaneously for LACC, a combination of radiation and cisplatin administered at a dose of 40 mg/m2 once a week for 6 weeks was applied.

CT images with 3-mm interslice distance to be used in EBRT for each patient were obtained using Toshiba (Toshiba Medical Systems) device. The data obtained from CT were transferred to the treatment planning system (TPS) (Eclipse v.15.1. Varian Medical Systems, Inc, Palo, CA, USA). The BRT phase was started 10–15 days after the EBRT was completed. For CT imaging to be used in BRT treatment planning, a urinary catheter of appropriate size is placed in the bladder and a Foley catheter balloon is inflated with 7 cc radiopaque saline. Then, TOs are placed on the patient. After the ovoids and tandem are properly fixed to each other, packing process is started. Packing is the fixation of gauze prepared with radiopaque material into the vagina. After the procedures of the patients were completed, CT images were obtained with a 3-mm interslice distance and transferred into TPS.

### Contouring

For EBRT, target volumes and OARs were delineated in the contouring system according to the recommendations of the Radiation Therapy Oncology Group (RTOG) [15]. Clinical target volumes (CTV) were contoured as CTV primer (CTV_P_) and CTV lymph nodes (CTV_LN_). CTV_P_ included gross target volumes (GTV), entire uterus, parametrium, and whole cervix and ≥2 cm of the vagina below the tumors. CTV_LN_ included involved nodes and relevant draining nodal groups (common iliac, internal and external iliac nodes, obturator and presacral nodes ). Planning target volume (PTV) margin was 0.5 cm in anterior-posterior direction and lateral direction, and 1 cm in cranio-caudal direction.

For BRT, target volumes and OARs were contoured based on GEC-ESTRO. CTV_HR_ created by contouring GTV at the time of brachytherapy, cervix, and suspected residual disease in clinical examination. CTV_IR_ included a margin of 5 mm given from the anterior and posterior and 10 mm from other directions around CTV_HR_.

### External beam radiotherapy

For each patient, treatment plans were generated using Varian DHX linear accelerator, which is capable of delivering both 3DCRT and IMRT with 80-leaf MLC system installed in the department of Radiation Oncology at XX University. For 3DCRT, plans were performed utilizing four-field box technique for 18 MV photons. 3DCRT treatment plans were generated using one anterior, one posterior, and two lateral fields. Beams were optimally weighted to provide the best PTV coverage. For IMRT plans, seven non-coplanar fields (0°, 52°, 104°, 156°, 204°, 256°, and 308°) were created using sliding window technique. IMRT plans were generated 6 MV photons. Anisotropic Analytical Algorithm (AAA) dose distributions were calculated after optimization with inverse planning with a grid of 2.5 mm. The prescribed dose, which was defined as the mean dose in the CTV, was 50.4 Gy in 28 fractions at 1.8 Gy per fraction. Plans were normalized as at least 95% of the CTV was required to be covered by at least 95% isodose of 50.4 Gy.

### Brachytherapy

For HDR-BRT boost plans were generated for each fraction using BrachyVision 15.1 (Varian Medical Systems, Inc, Palo, CA, USA). MO and IO treatment plans were generated. MO treatment plans were created by varying the dwell times using trial and error method until the optimum plan was obtained. IO was used to generate an inverse plan, which identifies the combination of dwell times that best conforms to dose constraints of CTV_HR_ and OARs. No manual optimization was allowed. The dwell times and dose constraints were changed until an optimal plan was obtained that meets the dose objective parameters of both target volume and OARs. Two different plans were created with dose prescriptions of 7 Gy × 4 fractions to CTV_HR_. The patients were created with HDR-BRT 7Gy × 4=28 Gy resulting into a prescription dose to the CTV_HR_.

### Dosimetric details

After treatment plans, EBRT and HDR-BRT boost plans were converted to EQD_2_ using a linear-quadratic model with an α/β of 3 for normal tissues and an α/β of 10 for tumor. EQD_2_ doses were then transferred to the primary CT for dose composite and DVH parameters analysis. The OARs D_2cm3_ were the main focus in this study for the purpose of following ICRU-report89 recommendations [11].

$$ {\mathrm{EQD}}_2=\frac{\mathrm{ND}\ \left(1+\mathrm{g}\ \frac{d}{\upalpha /\upbeta}\right)}{\left(1+\frac{2}{\upalpha /\upbeta}\right)} $$

Here *N*, *d*, and *g* represent a fraction number, a dose per fraction, and an incomplete repair function that is 1 for HDR. Afterward, the DVH parameters (e.g., D_2cm3_) in EQD_2_ were added for each EBRT and HDR plan. Both approaches can be simply done using an Excel spreadsheet (Microsoft Corporation, Redmond, WA, USA) available as a template on the American Brachytherapy Society (ABS). D_90_, D_95_, and D_98_ were compared for the CTV_HR_ total EQD2 between all treatment plans. The EQD_2_ for 2 cm^3^, 1 cm^3^, and 0.1 cm^3^ of rectum, bladder, small bowel, and sigmoid were calculated for all plans.

### Statistical analysis

All statistical analysis were performed using the Statistical Package for Social Sciences (SPSS) version 25.1 (SPSS, Chicago, IL, USA). The one-way ANOVA test was used to evaluate the treatment techniques and the Tukey test was used to determine the group that caused the difference. A *p* value of ˂0.05 was considered to be significant.

## Results

**Fig. 1:**
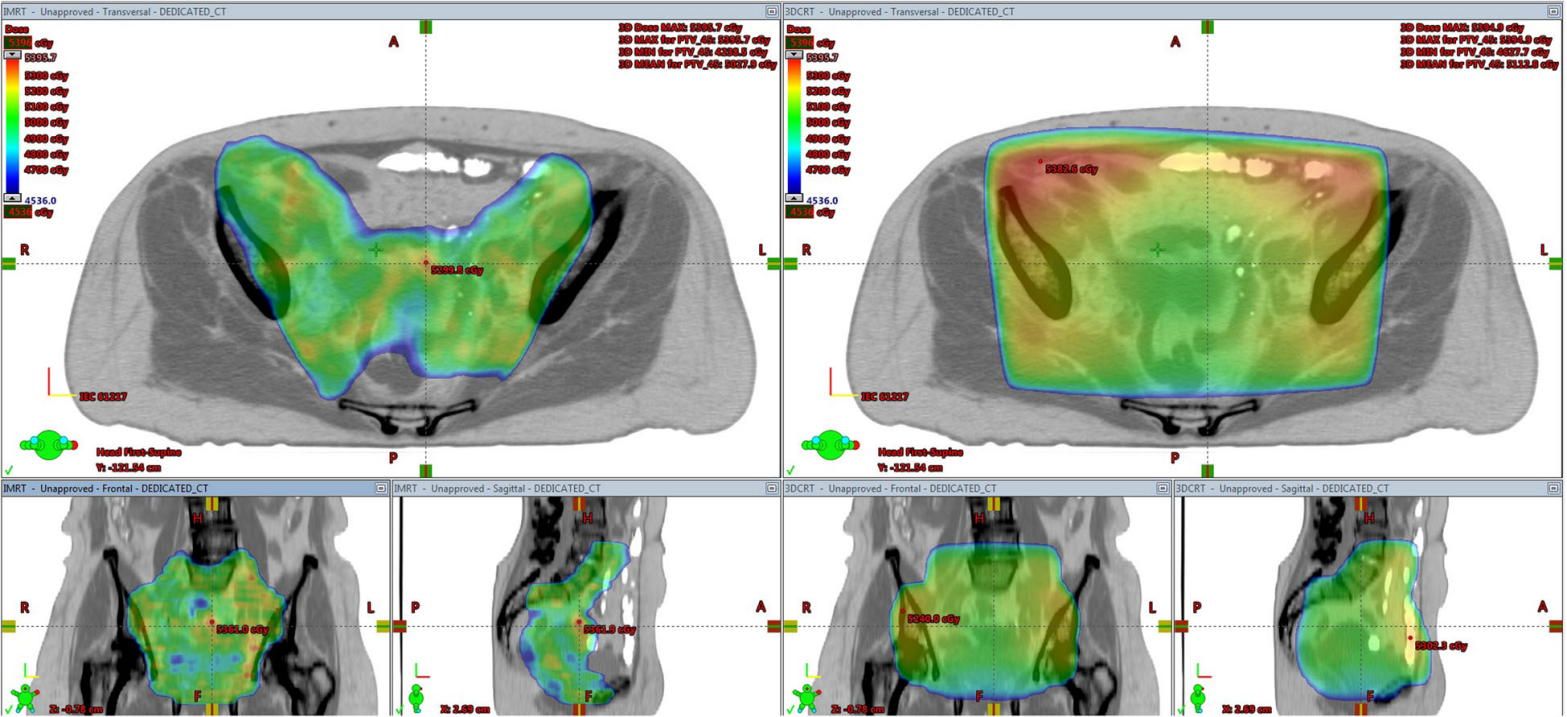
The comparison of dose distribution between IMRT plan and 3DCRT plan

**Fig. 2:**
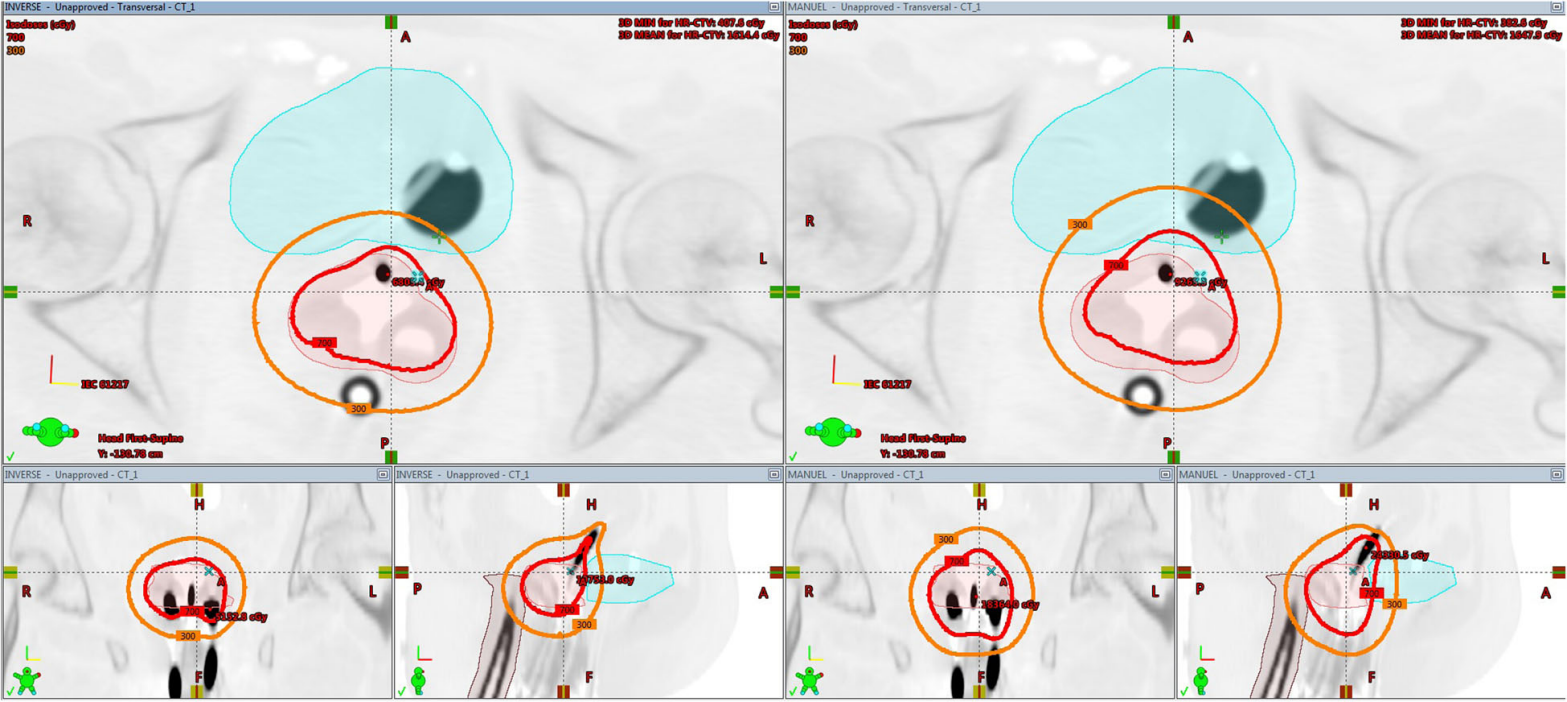
The comparison of dose distribution between IO plan and MO plan

**Fig. 3:**
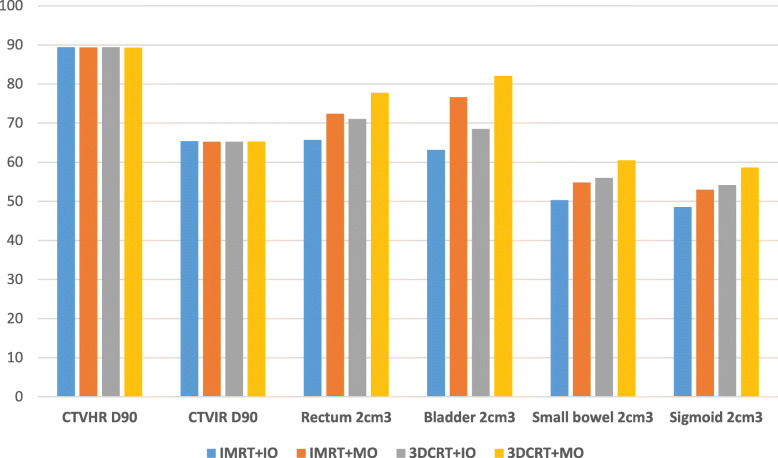
The mean equieffective dose in 2 Gy per fraction (EQD_2_) of target volume

In Table 1, the comparison of the total EQD_2_s of the groups formed from different treatment plans for target volumes is given. Figure 1 shows the evaluation of the 3DCRT plan with IMRT plan, and Fig. 2 shows the evaluation of the MO plan with IO plan. Any significant difference was not seen between the treatment groups in terms of CTV_HR_ D_90_ (*p* = 0.908) and CTV_IR_ D_90_ (*p* = 0.855); However, in IMRT + IO technique, more improved CTV_HR_ D_95_ (*p* = 0.000) and CTV_HR_ D_98_ (*p* = 0.000) values were obtained. Table 2 shows the comparison of the total EQD_2_ values achieved with different techniques used for OARs. A comparison of EQD_2_ doses for reference values of treatment plans are shown in Fig. 3.

**Table 1:** The total EQD2 of target volume for different treatment technique

Parameters	IMRT+IO	IMRT+MO	3DCRT+IO	3DCRT+MO	*p*-value*
CTV_HR_D_98_ (Gy)	77.56±2.18	75.33±0.99	77.55±1.90	75.32±1.16	**0.000**
CTV_HR_ D_95_ (Gy)	82.21±1.26	80.96±0.80	81.98±1.20	80.73±0.86	**0.000**
CTV_HR_ D_90_ (Gy)	89.42±0.17	89.36±0.48	89.40±0.17	89.33±0.48	0.908
CTV_IR_ D_90_ (Gy)	65.34±0.29	65.27±0.41	65.24±0.38	65.29±0.34	0.855

*IMRT* intensity-modulated radiation therapy, *3DCRT* three-dimensional conformal radiation therapy, *IO* inverse optimization, *MO* manual optimization

**Table 2:** The total EQD_2_ of organ at risks for different treatment technique

Parameters		IMRT+IO	IMRT+MO	3DCRT+IO	3DCRT+MO	*p*-value*
Rectum	D_2cm_^3^ (Gy)	65.70±9.29	72.37±5.32	71.08±9.46	77.75±6.21	**0.001**
	D_1cm_^3^ (Gy)	65.96±4.54	75.32±4.80	71.53±4.93	81.89±5.65	**0.000**
	D_0.1cm_^3^ (Gy)	76.51±6.78	88.38±5.97	82.35±6.70	84.22±6.12	**0.000**
Bladder	D_2cm_^3^ (Gy)	63.10±5.52	76.65±9.89	68.48±6.17	82.03±9.74	**0.000**
	D_1cm_^3^ (Gy)	67.01±7.03	81.40±11.67	72.03±7.37	86.41±11.21	**0.000**
	D_0.1cm_^3^ (Gy)	76.52±10.68	102.25±20.78	81.03±11.05	106.76±20.35	**0.000**
Small bowel	D_2c_^m3^ (Gy)	50.27±2.67	54.76±4.96	56.01±2.15	60.50±4.80	**0.000**
	D_1cm_^3^ (Gy)	51.93±3.88	58.52±12.05	57.53±3.46	64.12±11.84	**0.005**
	D_0.1cm_^3^ (Gy)	54.50±5.22	64.90±20.66	59.69±4.86	70.10±20.45	**0.037**
Sigmoid	D_2cm_^3^ (Gy)	48.52±2.86	53.00±3.27	54.14±3.69	58.62±4.12	**0.000**
	D_1cm_^3^ (Gy)	50.19±3.90	55.37±4.12	55.49±4.75	60.67±5.00	**0.000**
	D_0.1cm_^3^ (Gy)	53.56±6.78	59.01±6.13	58.02±7.22	63.47±6.90	0.002

*IMRT* intensity-modulated radiation therapy, *3DCRT* three-dimensional conformal radiation therapy, *IO* inverse optimization, *MO* manual optimization

Although there was a significant difference in the D_2cm_^3^ (*p* = 0.001), D_1cm_^3^ (*p* = 0.000), and D_0.1cm_^3^ (*p* = 0.000) values of the rectum, a significant difference was found in the IMRT + IO technique compared to other techniques. Considering the D_2cm_^3^ (*p* = 0.000), D_1cm_^3^ (*p* = 0.000), and D_0.1cm_^3^ (*p* = 0.000) values of the bladder, IMRT+IO technique was found to be statistically significant compared to other techniques. When the D_2cm_^3^ (*p* = 0.000), D_1cm_^3^ (*p* = 0.005), and D_0.1cm_^3^ (*p* = 0.037) values of the small intestine were examined, a significant difference was found between the IMRT+IO technique and the other techniques. Statistical comparisons of the techniques for CTVs and OARs are given in Tables 3 and 4.

**Table 3:** Comparison of statistical significance for target volume

Parameters	IMRT+IO vs. IMRT+MO	IMRT+IO vs. 3DCRT+IO	IMRT+IO vs. 3DCRT+MO	IMRT+MO vs. 3DCRT+IO	IMRT+MO vs. 3DCRT+MO	3DCRT+IO vs. 3DCRT+MO
CTV_HR_D_98_	**0.002**	1.000	**0.002**	**0.003**	1.000	**0.002**
CTV_HR_ D_95_	**0.009**	0.930	**0.002**	**0.046**	0.930	**0.009**
CTV_HR_ D_90_	0.963	0.997	0.901	0.993	0.997	0.963
CTV_IR_ D_90_	0.937	0.996	0.854	0.983	0.996	0.937

*IMRT* intensity-modulated radiation therapy, *3DCRT* three-dimensional conformal radiation therapy, *IO* inverse optimization, *MO* manual optimization

**Table 4:** Comparison of statistical significance for OARs

Parameters		IMRT+IO vs. IMRT+MO	IMRT+IO vs. 3DCRT+IO	IMRT+IO vs. 3DCRT+MO	IMRT+MO vs. 3DCRT+IO	IMRT+MO vs. 3DCRT+MO	3DCRT+IO vs. 3DCRT+MO
Rectum	D_2cm_^3^	0.100	0.244	**0.000**	0.969	0.244	0.100
	D_1cm_^3^	**0.000**	**0.004**	**0.000**	0.174	**0.004**	**0.000**
	D_0.1cm_^3^	**0.000**	0.071	**0.000**	0.059	0.071	**0.000**
Bladder	D_2cm_^3^	**0.000**	0.274	**0.000**	**0.037**	0.274	**0.000**
	D_1cm_^3^	**0.001**	0.483	**0.000**	**0.046**	0.483	**0.001**
	D_0.1cm_^3^	**0.000**	0.876	**0.000**	**0.005**	0.876	**0.000**
Small bowel	D_2cm_^3^	**0.012**	**0.001**	**0.000**	0.814	**0.001**	**0.012**
	D_1cm_^3^	0.185	0.316	**0.002**	0.990	0.316	0.185
	D_0.1cm_^3^	0.238	0.778	**0.030**	0.776	0.778	0.238
Sigmoid	D_2cm_^3^	**0.005**	**0.000**	**0.000**	0.809	**0.000**	**0.005**
	D_1cm_^3^	**0.013**	**0.010**	**0.000**	1.000	**0.010**	**0.013**
	D_0.1cm_^3^	0.130	0.276	0.001	0.978	0.276	0.130

*IMRT* intensity-modulated radiation therapy, *3DCRT* three-dimensional conformal radiation therapy, *IO* inverse optimization, *MO* manual optimization

## Discussion

In this study, the dosimetric advantages of IMRT + IO, IMRT + MO, 3DCRT + IO, and 3DCRT + MO techniques for cervical cancer were compared. In our study, CTV_HR_ and CTV_IR_ total EQD_2_ doses for all plans were 89 Gy and 65 Gy, respectively. Recommended dose limitations for bladder, small intestine, and sigmoid were provided in all techniques. It was observed that the ≤ 75 Gy recommendation for the rectum was provided by IMRT + IO, IMRT + MO and 3DCRT + IO techniques, but not by 3DCRT + MO technique.

Dimopoulos et al. compared DVH in 141 cervical cancer patients they evaluated in 2008. In the first stage, they delivered a total of 45–50.4 Gy of EBRT from 1.8 Gy in 25–28 fractions to the study population. In the second stage, they delivered a total of 28 Gy BRT in 4 fractions of 7 Gy. They targeted 84–89 Gy as the total EQD_2_ dose. Dimopoulos et al. showed that patients with a CTV_HR_ D_90_ greater than 87 Gy achieved a local recurrence rate of approximately 4%, compared to 20% for patients with a CTV_HR_ D_90_ less than 87 Gy [16]. Our study was in good compliance with that of Dimopoulos et al. and 89.42 ± 0.17, 89.36 ± 0.48, 89.40 ± 0.17, and 89.33 ± 0.48 Gy were used for IMRT + IO, IMRT + MO, 3DCRT + IO, and 3DCRT + MO techniques, respectively.

Jamema et al. were compared MO and IO methods according to CTV_HR_ criteria. In practice, they used afterloading BRT device and tandem-ovoid applicators. In their study, they stated that there was no difference between the plans made with MO and IO for CTV_HR_ and that no statistical significance was found in the evaluation made for the D_90_ criteria [17]. In our study, when we compared optimization methods for CTV_HR_ D_90_ criterion, optimization methods were significantly better in the IO technique.

Kumar et al. investigated different HDR brachytherapy dose programs of EQD_2_ for CTV_HR_ and OARs in 50 cervical cancer patients, and compared their dosing schedules with those cited in the current literature. In their study, they defined different doses and dose fractions for BRT after delivery of a total of 45 Gy EBRT in 25 fractions. They evaluated their study for 7 Gy × 4 fr BRT delivered after 45 Gy EBRT, and found that the total EQD_2_ of CTV_HR_ D_90_ was 89.03 ± 2. In their study, they found that the doses used for the volumes of 2 cm^3^ of the rectum, bladder, and sigmoid received as 71.11 ± 60, 77.26 ± 90, and 55.44 ± 70 Gy, respectively. In addition, they found that the doses used for the volumes of 0.1 cm^3^ of the rectum, bladder, and sigmoid received as 88.65 ± 12, 93.22 ± 15, and 66.30 ± 14 Gy, respectively [18]. In our current study, the mean total EQD_2_ of CTV_HR_ D_90_ was found to be 89.37 ± 33 Gy in all techniques similar to the results obtained by Kumar et al. In our study, doses delivered in IMRT + IO, IMRT + MO, 3DCRT + IO, and 3DCRT + MO techniques for 2 cm^3^ of the rectum were 65.70 ± 9.29, 72.37 ± 5.32, 71.08 ± 9.46, and 77.75 ± 6.21 Gy, respectively. In our study in the IMRT + IO technique, 65.70 ± 9.29 Gy was delivered for 2 cm^3^ of the rectum which was more significant compared to the findings of Kumar et al. but this level of significance was not detected in other techniques. Total EQD_2_ for 2 cm^3^ of bladder and sigmoid in IMRT + IO, IMRT + MO, and 3DCRT + IO techniques was more significant compared to the data reported by Kumar et al.

In the study performed in 2011, Potter et al. delivered 50.4 Gy EBRT and 7 Gy × 4 fr BRT to 156 patients and evaluated their outcomes. They analyzed the clinical results and benefits of the protocol they applied. They found that the total EQD_2_ of the CTV_HR_ D_90_ was 93 Gy. They found total EQD_2_ s for 2 cm^3^ of rectum, bladder, and sigmoid volumes as 65, 86, and 64 Gy, respectively [19]. In this study, we found that the total EQD_2_ of CTV_HR_ of D_90_ was 89.37 Gy in all techniques. Similar to the findings of Potter et al., we found 65 Gy for 2 cm^3^ of the rectum in the IMRT + IO technique. According to Potter et al. for 2 cm^3^ of bladder and sigmoid volumes, better dose protection was provided in all techniques. In the current study, there have been several limitations. This is a dosimetric study, and it does not include vital aspects required for clinical use. The number of patients used for comparison was limited to 15; this may be improved in the next study to obtain a better sample.

## Conclusions

Based on the EQD_2_ in this study, there is a potential clinical advantage for IMRT+IO technique compared with IMRT+MO, 3DCRT+IO, and 3DCRT+MO for the treatment of cervical cancer. It is seen that IMRT+IO technique is statistically more advantageous against IMRT+MO, 3DCRT+IO and 3DCRT+MO technique in terms of critical organs. In the treatment of cervical cancer patients, it is recommended to prefer the IMRT+IO technique for the optimum dose value of time and OARs.

## Data Availability

The datasets used and analyzed during the current study are available from the corresponding author on reasonable request.

## Abbreviations

EQD_2_: Equieffective dose
DVH: Dose volume histograms
EBRT: External beam radiotherapy
HDR-BRT: High-dose rate brachytherapy
3D-CRT: Three-dimensional conformal radiotherapy
IMRT: Intensity-modulated radiotherapy
MO: Manual optimization
IO: Inverse optimization
LACC: Locally advanced cervical cancer
CT: Computed tomography
MRI: Magnetic resonance imaging
TO: Tandem-ovoid
FIGO: International Federation of Gynecology and Obstetrics
GEC-ESTRO: European Society for Radiotherapy & Oncology
TPS: Treatment planning system
CTV_HR_: Clinical target volume
GTV: Gross target volumes
PTV: Planning target volume
AAA: Anisotropic Analytical Algorithm
